# Identifying pathogenicity-related genes in the pathogen *Colletotrichum magnum* causing watermelon anthracnose disease *via* T-DNA insertion mutagenesis

**DOI:** 10.3389/fmicb.2023.1220116

**Published:** 2023-07-20

**Authors:** Zhen Guo, Huijie Wu, Bin Peng, Baoshan Kang, Liming Liu, Chaoxi Luo, Qinsheng Gu

**Affiliations:** ^1^National Key Laboratory for Germplasm Innovation and Utilization of Horticultural Crops, Zhengzhou Fruit Research Institute, Chinese Academy of Agricultural Sciences, Zhengzhou, China; ^2^National Key Laboratory for Germplasm Innovation and Utilization for Fruit and Vegetable Horticultural Crops, College of Plant Science and Technology, Huazhong Agricultural University, Wuhan, China

**Keywords:** fungal transformation, ATMT, T-DNA integration, virulence gene, *Colletotrichum magnum*

## Abstract

Fruit rot caused by *Colletotrichum magnum* is a crucial watermelon disease threatening the production and quality. To understand the pathogenic mechanism of *C. magnum*, we optimized the *Agrobacterium tumefaciens*-mediated transformation system (ATMT) for genetic transformation of *C. magnum*. The transformation efficiency of ATMT was an average of around 245 transformants per 100 million conidia. Southern blot analysis indicated that approximately 75% of the mutants contained a single copy of T-DNA. Pathogenicity test revealed that three mutants completely lost pathogenicity. The T-DNA integration sites (TISs) of three mutants were Identified. In mutant Cm699, the TISs were found in the intron region of the gene, which encoded a protein containing AP-2 complex subunit σ, and simultaneous gene deletions were observed. Two deleted genes encoded the transcription initiation protein SPT3 and a hypothetical protein, respectively. In mutant Cm854, the TISs were found in the 5′-flanking regions of a gene that was similar to the *MYO5* encoding Myosin I of *Pyricularia oryzae* (78%). In mutant Cm1078, the T-DNA was integrated into the exon regions of two adjacent genes. One was 5′-3′ exoribonuclease 1 encoding gene while the other encoded a WD-repeat protein retinoblastoma binding protein 4, the homolog of the MSl1 of *Saccharomyces cerevisiae*.

## Introduction

*Colletotrichum magnum*, is one of the major pathogens causing watermelon anthracnose (Rossman et al., 2016; Damm et al., 2019; Guo et al., 2022). Recently, it was reported on *Cucumis sativus*, *Capsicum* spp., *Lagenaria siceraria*, *Lobelia chinensis*, and *Luffa cylindrica* in China (Tsay et al., 2010; Li et al., 2013; Liu F. et al., 2022). *C. magum*, whoes conidia were larger than those of other *Colletotrichum* species, belongs to *C. magum* species complex (Damm et al., 2019). When *Colletotrichum* species infect the host, they establish biotrophic and necrotrophic lifestyles (Munch et al., 2008). To colonize host tissues, pathogens of *Colletotrichum* species generally form appressoria which penetrate the epidermis of the host using penetration pegs. The formation of appressoria usually begins with the germination of conidia (Tucker and Talbot, 2001). In the biotrophic phase of the pathogen, the primary hyphae infect the epidermal and mesophyll cells and do not cause macroscopically visible damage to the host (De Silva et al., 2017). Subsequently, when differentiation of the primary hyphae produces secondary hyphae that spread throughout host tissue, the necrotrophic infection phase occurs (Wharton et al., 2001; Moraes et al., 2015).

The taxonomy, population structure, and disease epidemiology of *C. magnum* have been extensively studied (Damm et al., 2019). However, little is known about the pathogenicity of *C. magnum*. *Agrobacterium tumefaciens*-mediated transformation (ATMT) allows the study of numerous fungi to discover candidate pathogenic genes (Tsuji et al., 2003; Lawrence et al., 2010; Zhang et al., 2013; Martinez-Cruz et al., 2017; Li et al., 2019; Binh et al., 2021; Chauhan et al., 2021). Compared to conventional transformation methods, this approach provides a high percentage of T-DNA single-copy integration (De Groot et al., 1998; Münch et al., 2011) and is suitable for random insertional mutagenesis. The T-DNA integration sites (TISs) of transformants labeled with T-DNA can be easily identified (Liu et al., 1995; Sun et al., 2019).

The molecular determinants of fungal pathogenicity remain to be clarified and require more attention. Depending on the method of plant infection and the genome size of the fungus, fungi presumably containing 60–360 virulence genes (Idnurm and Howlett, 2001). Some genes that play important roles in signal cascades, cell wall degradation, the formation of infection structures, respond to the host environment, participat in the synthesis of toxins, and avoid and overcom plant defenses, have been identified as virulence or pathogenicity genes (Idnurm and Howlett, 2001; Werner et al., 2007; Gong et al., 2022; Jiao et al., 2022). To form an infection structure, fungi secrete effector molecules, such as the mutant of *C*. *higginsianum* is deficient in dihydroxynaphthalene melanin metabolism and is unable to form mature and infectious appressoria, thereby exhibiting reduced pathogenicity (Liu et al., 2013). In *C*. *gloeosporioides* and *Magnaporthe grisea*, the protein encoded by the protein kinase A is necessary for the mature appressorium to mediated plant infection (Gilbert et al., 2006; Cai et al., 2013). In *C. lindemuthianum*, the transcriptional activator gene (*CLTA1*) is disrupted, and mutant H433 cannot form necrotrophic secondary hyphae (Dufresne et al., 2000). Furthermore, allantoicase genes may participate in appressorium formation in *C. graminicola* (Münch et al., 2011).

In the present study, we established a stable ATMT protocol for *C. magnum* and generated a library of hygromycin B transformants. In the virulence assays on leaves, three mutants with defective pathogenicity were generated. The TISs of the three mutants were identified. Six putative pathogenic genes were identified.

## Materials and methods

### Fungus and plasmid preparation

The *C. magnum* wild-type (WT) strain CAASZK4 was isolated from watermelons in Kaifeng City, Henan Province, China (Guo et al., 2022). The strain AGL-1 of *A tumefaciens*, containing plasmid pATMT1 which carries a hygromycin B resistance cassette, was used as a T-DNA donor for fungal transformation (Zheng et al., 2011; Li et al., 2019).

### ATMT of *Colletotrichum magnum*

The WT strain (CAASZK4) were grown on potato dextrose agar (PDA) medium complemented with a range of hygromycin B concentrations (0, 20, 40, 60, 80, 100, 150, 200, and 250 mg/L) and were incubated at 27°C in darkness for 7 d.

ATMT-based transformation of *C. magnum* was performed according to the protocol described by Cai et al. with slight modifications (Cai et al., 2013). The AGL-1 strain grew at 28°C in 5 mL of Luria–Bertani (LB) medium supplemented 25 mg/L rifampicin and 50 mg/L kanamycin. Cultures were centrifuged again. Finally, the cells were resuspended in induction medium (IM, Bundock et al., 1995) supplemented with acetosyringone (AS, 200 μM), and incubated for 6 h to achieve OD600 nm values (0.6–0.8).

*C. magnum* grew on synthetic nutrient-poor agar medium (SNA, Nirenberg, 1976) for 25 d at 27°C in the dark. Subsequently, the conidia of *C. magnum* were cleaned thrice sterile water and diluted in IM to achieve different concentrations (1 × 10^6^–1 × 10^8^ spores/ml). Aliquots of 100 μL of bacterial culture and 100 μL of conidial suspension were plated onto the 0.45 μm pore nitrocellulose filter of the co-cultivation medium (CM) containing 200 μM AS. After cocultivation for 84 h at 23°C in the dark, the membranes were transferred to PDA containing 200 mg/L cefotaxime, 100 mg/L timentin, and 80 mg/L hygromycin B, and cultured for 18 d at 27°C in the dark. Mycelial plugs from the edges where fresh mycelia were cultured on PDA containing 80 mg/L hygromycin B were used to confirm resistance. To determine the mitotic stability of the transformants, 40 randomly selected transformants were cultured on PDA without hygromycin B for four generations and then transferred back to PDA with hygromycin B.

### DNA extraction and sequence analysis

The transformants and the WT strains of *C. magnum* were transferred to 100 mL fresh liquid PDA and cultured for 20 d at 27°C in the dark. Genomic DNA of 40 transformants and the WT strains of *C. magnum* were extracted by CTAB (Sangon Biotech, China).

To analyze genome integration of the transferred genes, the *hph* gene was amplified using the primer pair hph-F + hph-R (Table 1) and sequenced. PCR was were performed according to the protocol described by Guo et al. with slight modifications (Guo et al., 2022). The annealing temperature of *hph* was 60°C. PCR amplicons were purified and sequenced by Sangon Biotech Company (Shanghai, China).

**Table 1:** Primer list used in TAIL-PCR.

Name	Sequence (5′ → 3′)	Reference
hph-F	ATTGAAGGAGCATTTTTTGGGC	This study
hph-R	CTATTCCTTTGCCCTCGGAC	This study
L1	GGGTTCCTATAGGGTTTCGCTCATG	Mullins et al. (2001)
L2	CATGTGTTGAGCATATAAGAAACCCT	Mullins et al. (2001)
L3	GAATTAATTCGGCGTTAATTCAGT	Mullins et al. (2001)
R1	GGCACTGGCCGTCGTTTTACAAC	Mullins et al. (2001)
R2	AACGTCGTGACTGGGAAAACCCT	Mullins et al. (2001)
R3	CCCTTCCCAACAGTTGCGCA	Mullins et al. (2001)
AD1^a^	NGTCGASWGANAWGAA	Liu et al. (1995)
AD2	NTCGASTWTSGWGTT	Liu et al. (1995)
AD3	TGWGNAGSANCASAGA	Sessions et al. (2002)

**Figure 1:**
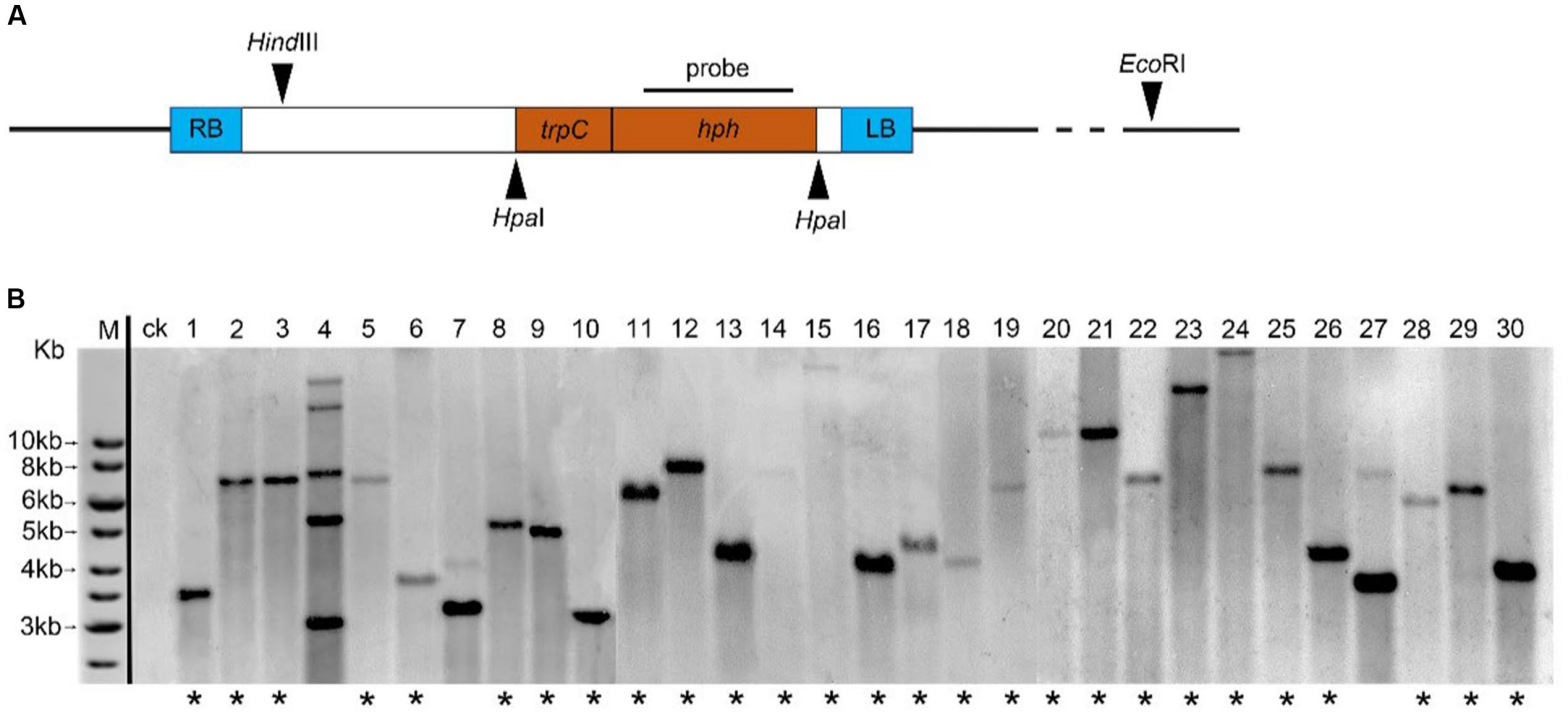
Analysis of T-DNA copy number in *C. magnum* transformants generated by *Agrobacterium tumefaciens*-mediated transformation (ATMT). **(A)** Probe used for hybridization, *Hind*III and *Eco*RI restriction sites are indicated; **(B)** Genomic Southern blot analysis of 30 transformants of *C. magnum* (1–30) generated by ATMT. Genomic DNA was restricted with *Hind*III and *Eco*RI. One band (lanes marked with an asterisk) represents a single T-DNA integration event 20, 22, and 28 represent genomic Southern blot analysis of Cm699, Cm854, and Cm1078, respectively.

The digestion of genomic DNA from 40 transformants or WT strains with *Eco*RI and *Hind*III (NEB, Ipswich, United States), electrophoresised on 0.75% agarose, were depurated, and transferred to nylon membranes (Hybond-N+; General Electric Company, Boston, United States). In the hybridization experiments, the digoxigenin (DIG) labeled probe corresponded to 1,380 bp *hph* of T-DNA from plasmid pATMT1 (Figure 1), and the *hph* gene was amplified using the primers hph-F and hph-R. Hybridization and chemiluminescence detection of the hybridized Dig-labeled probes were performed using the DIG High Prime DNA Labeling and Detection Starter Kit (Roche, Germany).

### Pathogenicity assay and morphological characterization of transformants

Mycelial plugs (5 mm) from 1,460 transformants and WT strains of *C. magnum* were cultured in PDA at 27°C in the dark for 5–15 d. The pathogenicity of these transformants and WT strain was evaluated on healthy detached watermelon leaves (*Citrullus lanatus* cv. Hongheping, 5–6 true leaves), which were previously washed with tap water, surface-sterilized with 75% ethanol for 30 s, and rinsed thrice with sterile water. Mycelial discs (5 mm in diameter) were individually placed on the right half of the leaves. PDA blocks (5 mm in diameter) were placed on the left half of the same leaves as controls. All leaves were incubated in sealed sterile plastic plates at 27°C in the dark. Leaf symptoms were assessed at 4 d post-inoculation. All transformants and WT strains were tested in triplicates.

Mycelial plugs (5 mm) from transformants and WT strains were transferred on 60 mm Petri dishes containing SNA medium, and incubated in the dark at 27°C for 25 d. The mycelia, conidia, and appressoria were observed under a light microscopy (Olympus BX51, Japan). The conidia formed in 60-mm Petri dishes were cleaned thrice with sterile water and resuspended in 1 mL sterile water. The number of conidia was calculated using a hemocytometer. To induce the formation of appressoria, the conidia were suspended in 1% glucose and incubated at 27°C in the dark. After 48 h, the germination rate of the conidia was measured. All transformants and WT strains were tested in triplicates.

### Cloning and sequencing of flanking T-DNA sequences

The T-DNA flanking sequences inserted into the genome were cloned using thermal asymmetric interlaced PCR (TAIL-PCR) protocol with minor modification (Liu et al., 1995; Mullins et al., 2001). Right border primers (R1, R2, and R3) and left border primers (L1, L2, and L3) and arbitrary degenerate primers (AD1, AD2, and AD-3) were utilized for TAIL-PCR (Table 1). PCR conditions were set as described by Mullins and Sessions (Mullins et al., 2001; Sessions et al., 2002), with minor modifications (Supplementary Table S1). The third TAIL-PCR products of all transformants displaying the highest brightness were purified using the FastPure Gel DNA Extraction Mini Kit (Vazyme, China). This fragment was ligated to the vector pTOPO-Blunt, which was transferred into *Escherichia coli* strain Top10 using a CV16-Zero Background pTOPO-Blunt Cloning Kit (Aidlab, China). *E. coli* harboring the pTOPO-Blunt vector was sequenced by Sangon Biotech Company (Shanghai, China). To isolate the tagged genes, the sequences flanking the T-DNA of each transformant were used to search the local genome database of WT *C. magnum* by BLAST+.^1^ Gene structure was predicted using the *C. fioriniae*, *C. gloeosporioides*, *C. graminicola*, *C. higginsianum*, *C. orbiculare*, and *C. sublineola* by the FGENESH program.^2^

### Whole-genome resequencing and analyses

Four micrograms of high-quality genomic DNA from three transformants (Cm699, Cm854, and Cm1078) were extracted from fresh mycelia using CTAB (Sangon Biotech, China) and used to construct a sequencing library, following the manufacturer’s instructions (Illumina Inc.). Paired-end sequencing libraries with an insert size of approximately 200 or 400 bp were sequenced using an Illumina HiSeq 2,500, NovaSeq 6,000, or MiSeq sequencer, with a reading length of 150 bp. In total, more than 6 GB of sequence data were generated for each transformant. The upstream and downstream genome sequences (500 bp) of the TISs were obtained using a comprehensive approach called TDNAscan^3^ searches against the genome database of WT *C. magnum* CAASZK4 (Sun et al., 2019). Using the GFF3 file of WT *C. magnum* as a reference genome organism, TDNAscan annotated all identified T-DNA insertions. Sequence homology searches were performed using NCBI protein database.^4^

Primers were designed to validate the TISs, T-DNA integration patterns, and deleted genes of Cm699, (Supplementary Table S2).

## Results

### Establishing a transformant library of *Colletotrichum magnum* using ATMT

To determine the hygromycin B sensitivity of *C. magnum*, the CAASZK4 strain was incubated on PDA supplemented with various concentrations of hygromycin B. These results indicate that the growth of *C. magnum* was inhibited by 60 mg/L hygromycin (Supplementary Figure S1). To exclude the possibility of false-positive transformants, 70 mg/L was chosen for the subsequent selection of resistant transformants.

Using the modified ATMT protocol, we obtained a library containing 1,460 transformants of the watermelon pathogen *C. magnum*. On an average, 245 transformants were generated from 10^8^ conidia. Analysis of 40 randomly selected transformants harboring hygromycin B resistance revealed that all 40 transformants showed mitotically stable integrated T-DNA. These transformants, which were successively cultured for four generations on PDA medium without the selection marker hygromycin B, did not result in the loss of integrated T-DNA, as shown by the ability of all 40 transformants to grow on the screening medium containing hygromycin B and *hph* gene amplification analysis (Supplementary Figures S2, S3).

### T-DNA copy number variation of transformants

To effectively identify the T-DNA integration events in the mutant library, 40 randomly selected transformants and WT strains were subjected to genomic Southern blot hybridization. Genomic DNA digested using *Eco*RI and *Hind*III was detected with the digoxigenin (Dig)-labeled probe harboring 1,380 bp *hph* gene (Figure 1A). Of the tested transformants, 30 displayed single-site TISs, nine harbored two TISs, and one harbored more than two TISs. Representative selections of 30 transformants are shown in Figure 1B.

### Identification of transformants with reduced pathogenicity

**Figure 2:**
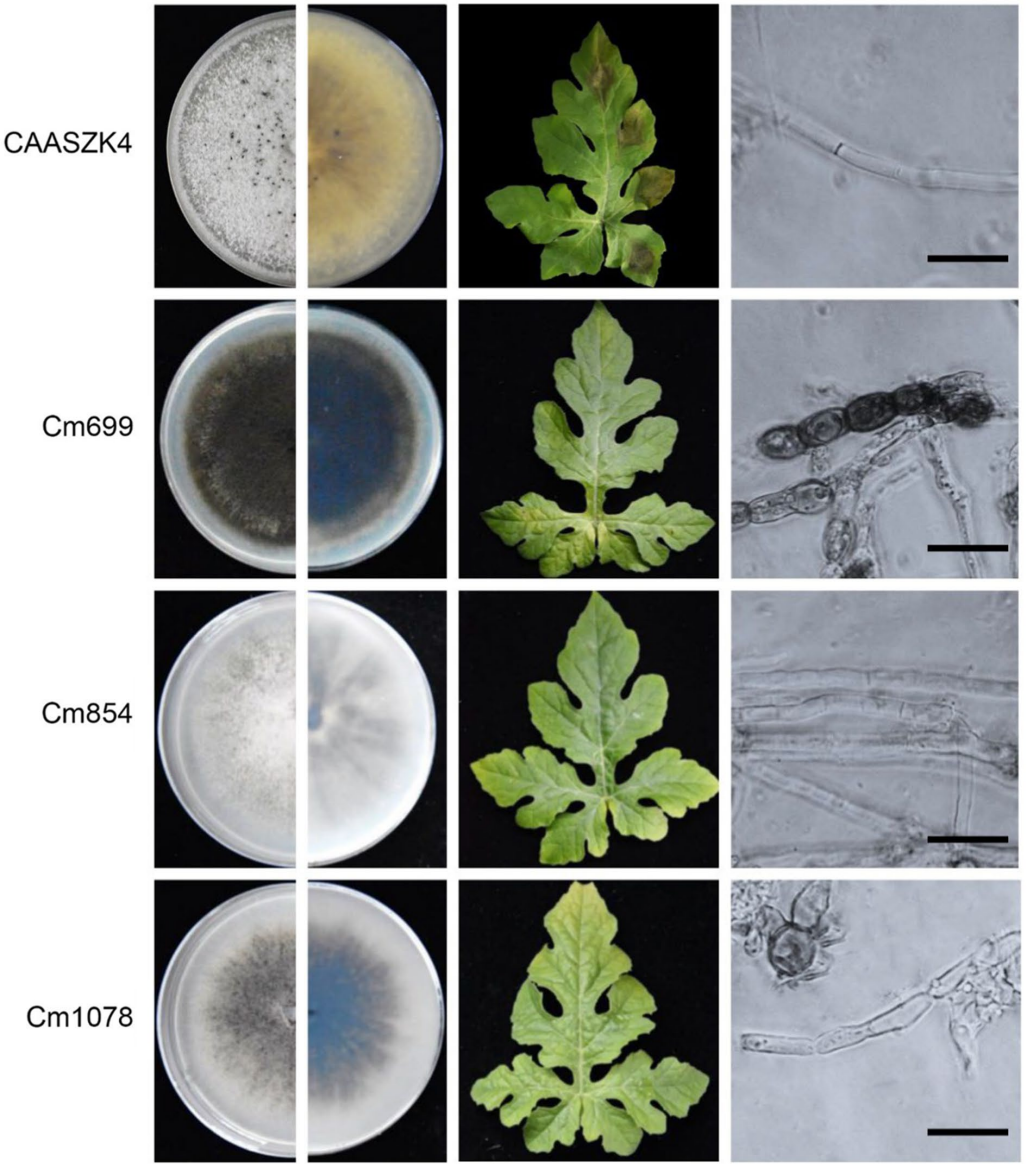
Phenotype and pathogenic development of *C. magnum* transformants produced by *Agrobacterium tumefaciens*-mediated transformation (ATMT). The phenotype during axenic growth on PDA is shown in the left panel. Pathogenicity assays on detached watermelon leaves (4 days post-inoculation (dpi)). Structure of *C. magnum* transformants mycelia. Bars represent 50 μm.

In total 1,460 transformants were analyzed for pathogenicity on detached leaves. Fourteen transformants were screened out for their strongly reduced virulence and further identified and analyzed on watermelon leaves. The three transformants of the 14 strains exhibited strongly reduced virulence in leaves assays. Interestingly, the three transformants (Cm699, Cm854, and Cm1078) caused no visible disease symptoms in watermelon leaves (Figure 2).

### Morphological and growth characterization of transformants with reduced pathogenicity

**Figure 3:**
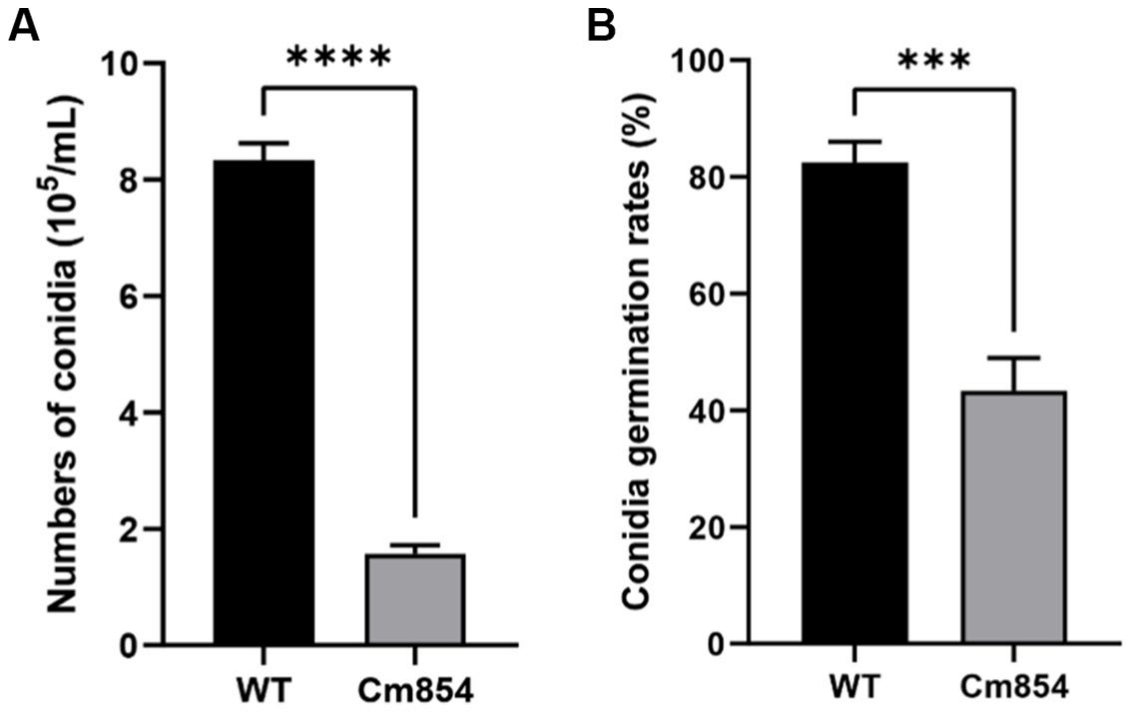
Numbers of conidia and the conidia germination rates of the wild-type (WT) strain CAASZK4 and of mutant Cm854. **(A)** Numbers of conidia of the wild-type (WT) strain and of mutant Cm854; **(B)** Conidia germination rates of the wild-type (WT) strain CAASZK4 and of mutant Cm854. Data were analysed with GraphPad Prism 9.0 (https://www.graphpad.com/). Asterisks over the error bars indicate the significant difference at the *p* = 0.05 level.

Three transformants (Cm699, Cm854, and Cm1078) exhibited significant morphology alterations compared to the WT strain (Figure 2). Of the three transformants, the hyphae of Cm699 showned severe deformity and pigment deposition compared to those of the WT strain (Figure 2); Cm699 and Cm1078 did not produce conidia, whereas Cm854 produced significantly fewer conidia than the WT strain (Figure 3A). In addition, the conidia germination rates of Cm854 were significantly lower than those of the WT strain (Figure 3B).

**Figure 4:**
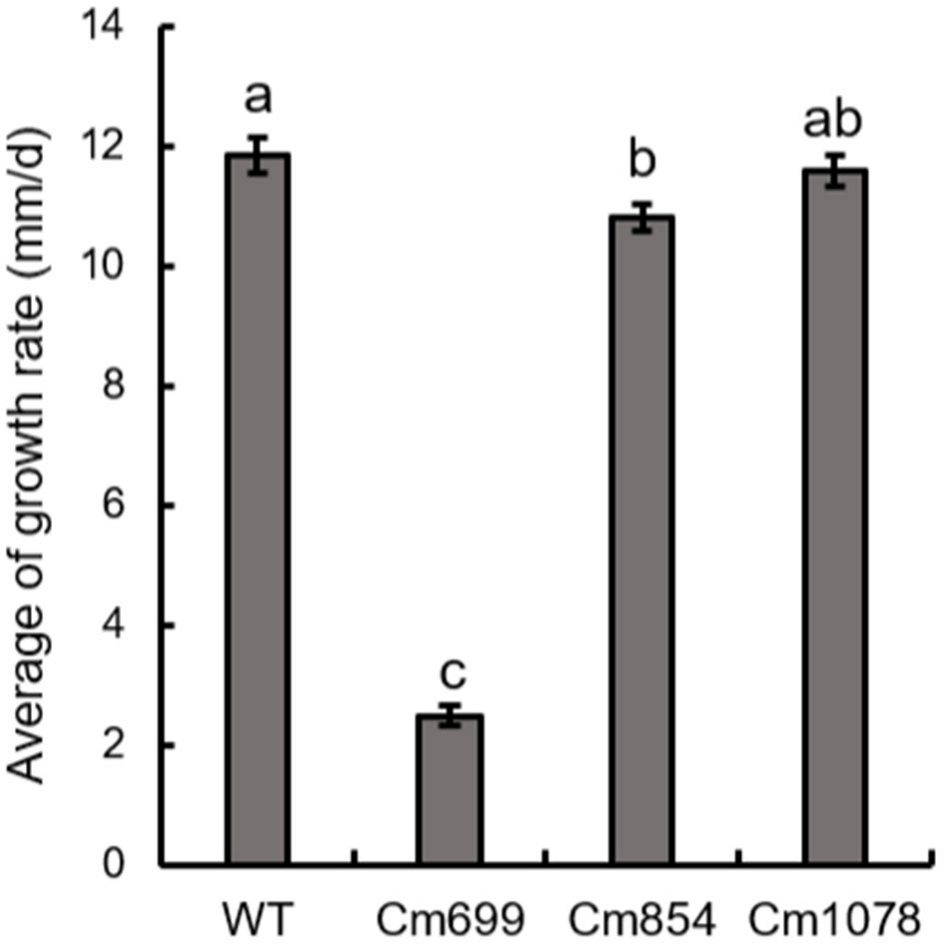
The growth rate of the three pathogenicity mutants and WT strains obtained in this study. Data were analysed with SPSS Statistics 19.0 (WinWrap Basic; http://www.winwrap.com) by one-way analysis of variance, and means were compared using Duncan’s test at a significance level of *p* = 0.05. Letters over the error bars indicate the significant difference at the *p* = 0.05 level.

The growth rates of Cm699 (2.5) and Cm854 (10.8) were significantly lower than those of the WT strain (11.9), and there was no significant difference between the Cm1078 (11.6) and WT strains (Figure 4).

### Identification of T-DNA insertion sites

According to genomic Southern blot experiments, three transformants (Cm699, Cm854, and Cm1078) harbored single T-DNA integrations (Figure 1B). To identify the genomic loci of the transformants where T-DNA was inserted, TAIL-PCR was performed on three pathogenic transformants. PCR fragments displayed more specific and higher intensity bands in the third round than those in the first and second of TAIL-PCRs (Supplementary Figure S5). Thus, PCR amplicons resulting from the third round of TAIL-PCR were cloned and sequenced. Three genomic DNA sequences flanking the left and right border of the T-DNA ranging from 0.7 to 3.0 kb were obtained, respectively. In two transformants (Cm699 and Cm1078), comparison of the sequences flanking the left and right border of the T-DNA with the genome sequence of WT *C. magnum* revealed significant similarities (Table 2). However, the sequence flanking the T-DNA of Cm854 showed no significant similarity with the genomic sequence of WT *C. magnum*. A analysis of T-DNA insertions demonstrated that two transformants (Cm699 and Cm1078) had interrupted protein-coding genes (Table 3).

**Table 2:** The sequences flanking left border and the right border of the T-DNA were aligned to the genome sequence of WT *C. magnum* by BLAST+.

Sequence	Chromosome	Position	Alignment length	Identify (%)	*e*-value
The left border of Cm699	Contig00001	4,448,487–4,450,001(−)	1,517	99.67	0
The right border of Cm699	Contig00001	4,438,996–4,439,880(−)	893	99.78	0
The right border of Cm1078	Contig00001	2,064,712–2,065,424(−)	715	99.02	0

**Table 3:** Summary of *C. magnum* genes identified from T-DNA flanking sequences.

Mutant	Open reading frame (ORF) predicted		Best BLAST match with functional annotation		
	T-DNA insertion^c^	Score	Putative function (NCBI accession no.)	Organism	*e*-value
Cm699-L^a^	1,583 bp upstream	35.71	Pro-apoptotic serine protease (XP_045270804.1)	*Colletotrichum gloeosporioides*	0
Cm699-R^b^	In ORF	53.30	AP-2 complex subunit σ(XP_045270800.1)	*Colletotrichum gloeosporioides*	7.00E-118
Cm1078-R	In ORF	32.45	histone-binding protein RBBP4 (XP_038750499.1)	*Colletotrichum karsti*	3.00E-108

a Genomic DNA sequences flanking the left border of the T-DNA.

b Genomic DNA sequences flanking the right border of the T-DNA.

c Putative position relative to open reading frame (ORF). Distance upstream of predicted start codon of predicted stop codon.

**Figure 5:**
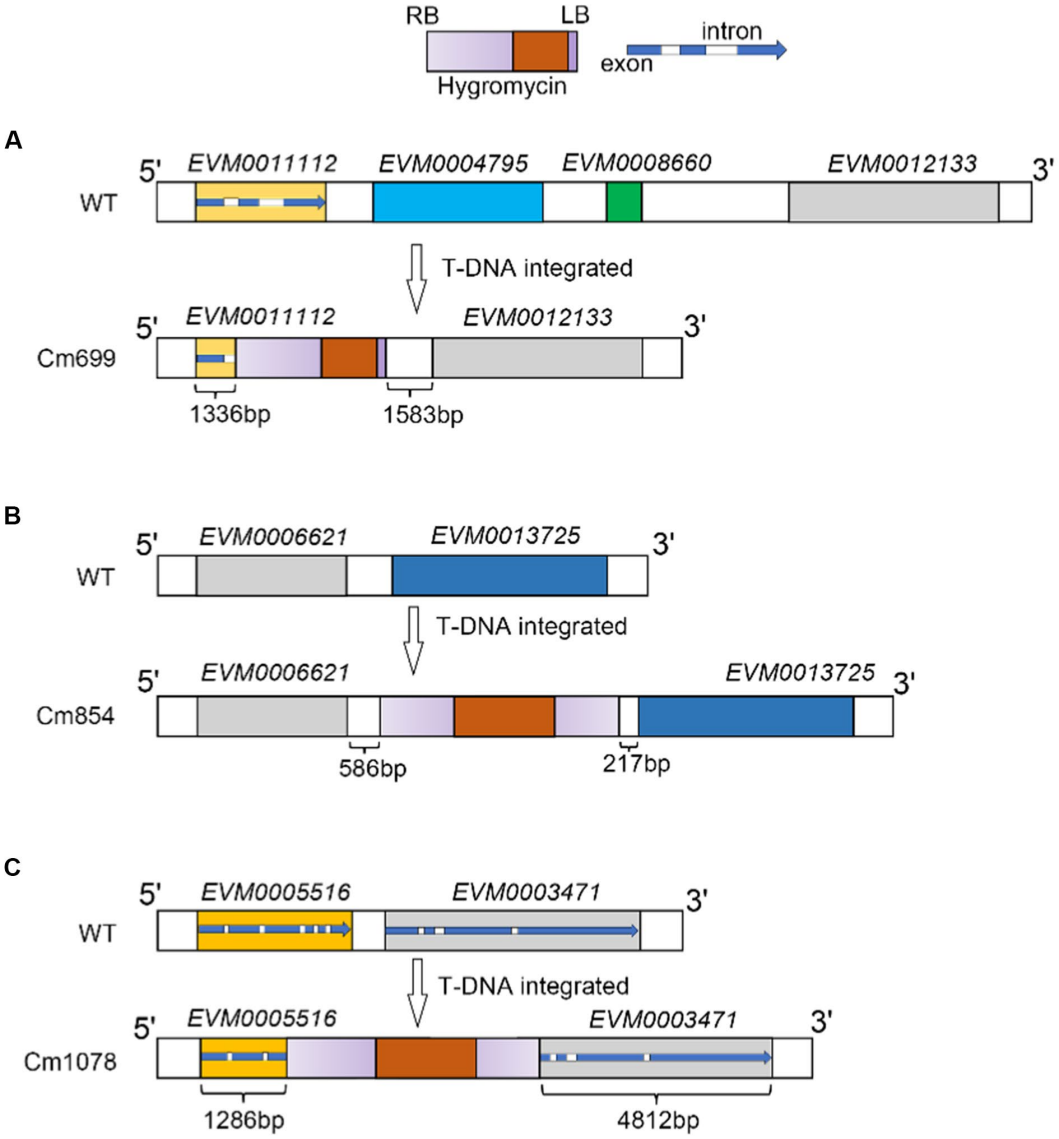
T-DNA integration sites patterns of T-DNA integration and in transformants Cm699, Cm854, and Cm1078. **(A)**, **(B)**, **(C)** Illustration of T-DNA integration into Cm699, Cm854, and Cm1078, respectively. The rectangle in gradient purple represents the ideal T-DNA between LB and RB. The rectangle in orange represents the T-DNA fragment coding the hygromycin resistance gene. In the blue arrow, the blue represents the exon as well as the white represents the intron. The integration sites were identified by whole genome re-sequencing in the text.

To further localize and characterize the TISs, whole-genome resequencing data from three transformants (Cm699, Cm854, and Cm1078) were utilized. Six T-DNA insertions in the three transformants were identified using TDNAscan (Table 4). However, all T-DNA insertions in the two transformants (Cm854 and Cm1078) were truncated (Table 5; Supplementary Figure S5). Furthermore, two T-DNA integration patterns, single-copy T-DNA with complete LB and RB and truncated tandem T-DNA repeats of LB or RB, were found in *C. magnum* (Figure 5). Using the local genome database of WT *C. magnum* as a reference genome, we found that T-DNA integration of transformant Cm699 occurred in the intron region starting 1,336-bp downstream of the start codon of the AP-2 complex subunit σ gene, and two genes were deleted, in the integration process. (Figure 5A). Based on whole-genome resequencing data, the two deleted genes were predicted to be the transcription initiation protein gene and hypothetical gene, respectively (Table 5). Furthermore, integration occurred within the promoter region 217-bp upstream of the start codon of the Myosin I gene in the transformant Cm854 (Figure 5B). In the Cm1078 transformant, the integration had in the exon region of two adjacent genes, one exon region starting 893-bp downstream of the start codon of WD-repeat protein retinoblastoma binding protein 4 gene (*RBBP4*), while the other exon region starting 4,812-bp upstream of the termination codon of 5′-3′exoribonuclease 1 gene (*XRN1*) (Figure 5C).

**Table 4:** T-DNA insertions identified by TDNAscan.

Mutant	Chromosome	Position	Informative reads	T-DNA truncation	Strand	Freq	Annotation
Cm699	Contig00001	4,439,884	CLR:8, DIR:0	tdna_st:-, tdna_end:282	−	1	intron
	Contig00001	4,448,487	CLR:18, DIR:3	tdna_st:6579, tdna_end:-	−	1	–
Cm854	Contig00005	7,323,116	CLR:55, DIR:0	tdna_st:-, tdna_end:282	−	1	–
	Contig00005	7,323,128	CLR:43, DIR:1	tdna_st:-, tdna_end:281	+	1	–
Cm1078	Contig00001	2,065,426	CLR:58, DIR:0	tdna_st:-, tdna_end:276	−	1	exon
	Contig00001	2,066,904	CLR:43, DIR:0	tdna_st:-, tdna_end:283	+	1	exon

**Table 5:** Summary of *C. magnum* genes identified from whole genome re-sequencing.

Mutant	Chromosome	Position	Annotation	Putative function
Cm699	Contig00001	4,438,548–4,440,264(−)	EVM0011112	AP-2 complex subunit σ
	Contig00001	4,441,377–4,445,085(−)	EVM0004795	Transcription initiation protein SPT3
	Contig00001	4,446,661–4,447,361(−)	EVM0008660	Hypothetical protein
	Contig00001	4,449,487–4,453,700(+)	EVM0012133	Pro-apoptotic serine protease
Cm854	Contig00005	7,319,862–7,322,530(+)	EVM0006621	Hypothetical protein
	Contig00005	7,323,345–7,327,847(+)	EVM0013725	Myosin I
Cm1078	Contig00001	2,064,140–2,066,069(−)	EVM0005516	Histone-binding protein RBBP4
	Contig00001	2,066,586–2,071,716(+)	EVM0003471	5′-3′ exoribonuclease 1

## Discussion

*C. magnum* is a crcucial fungus that induces watermelon anthracnose (Damm et al., 2019). Understanding the mechanisms of its pathogenesis will facilitate disease management. ATMT is an effective method for exploring pathogenicity associated genes in pathogenic fungi (Fitzgerald et al., 2003; Leclerque et al., 2004; White and Chen, 2006; Zhang et al., 2013; Li et al., 2019; Villena et al., 2020; Binh et al., 2021; Casado-Del Castillo et al., 2021; Chauhan et al., 2021; Liu et al., 2021). Howerever, this method has not been applied to *C. magnum*. In this study, we optimized the ATMT protocol for the stable transformation of *C. magnum*, yielding130–360 (average = 245) transformants per 10^8^ spores.

The mode and frequency of T-DNA insertions into the pathogenic genome are vital for the identification of disrupted genes. In *Magnaporthe oryzae*, not only single-copy T-DNA integration into genome, but multi-copy integration of the entire Ti-plasmid was found (Li et al., 2007). In this study, 75% of the transfromants had a single copy of T-DNA randomly located in the genome, whereas only approximately 2% multi-copy integration was found, which was consistent with previous reports (Münch et al., 2011). The transformants generated using the ATMT protocol, in combination with the identification of TISs, were used to determine the virulence factors of *C. magnum*, which can be applied in forward genetics.

Pathogenically defective transformants were predicted through the pathogenicity assay of the transformants, which were obtained using ATMT protocol for *C. magnum*. The penetration barriers (cuticle and epidermis) of the host are the main structures that defend against pathogenic infections. If both penetration barriers are destroyed and the quiescent infection is dispruted, the transformants are capable of colonizing the host tissues (Takano et al., 2000; Tsuji et al., 2003). Beacuse the defense reaction of young leaves is incomplete compared to that of old leaves when the host is infected by fungi, young leaves are more suitable for screening and assessing the pathogenicity of transformants (Cai et al., 2013). In this study, pathogenicity assay for all transformants was conducted on healthy and wounded watermelon leaves (5–6 true leaves), and the mycelium of the pathogen was used to inoculate watermelon leaves. Of these, compared with the WT strain, three transformants revealed no pathogenicity and 11 transformants showed impaired pathogenicity on healthy detached watermelon leaves (Figure 2; Supplementary Figure S6). It may be that potential pathogenic genes of three transformants affected the ability of the mycelium to infect the host.

As the integration of T-DNA into the genome of the pathogen affected the expression of pathogenic genes of pathogen, defective transformants were obtained. Hence, the TISs analysis was necessary. To identify the location and mode of the T-DNA insertions into the fungal genome, flanking sequence analysis was performed *via* TAIL-PCR (Huser et al., 2009; Liu et al., 2013). However, the application of TAIL-PCR for the identification of TISs was limited because the left and/or right ends of T-DNA can be truncated before insertion into the genome (Schouten et al., 2017). In this study, no LB-or RB-flanking sequences were cloned from the Cm854 transformants. For the Cm1078 transformants, LB flanking sequences were not cloned. This result may be explained by the truncation of LB, similar to observations in other fungi (Maruthachalam et al., 2008; Cai et al., 2013). Next-generation sequencing technologies have complemented the deficiency of TAIL-PCR for T-DNA site identification. The TISs of three transformants (Cm699, Cm854, and Cm1078) were successfully identified using whole-genome resequencing data (Table 5). Moreover, the sequences of 500 bp flanking TISs and the position of TISs in the genome of *C. magnum* were obtained using TDNAscan. Notably, the TISs of Cm699 and Cm1078 examined using TDNAscan were consistent with those obtained using TAIL-PCR (Tables 3, 5).

Some genes regulate infection-related morphogenesis during pathogenesis (Idnurm and Howlett, 2001). In the present study, six potentially pathogenic genes were identified. One potential pathogenicity gene encoded a protein containing the AP-2 complex subunit σ. AP-2 complexes were composed of four subunits: two large subunits (β2 and α), a medium subunit μ, and a small subunit σ (Liu C. et al., 2022). In *S. cerevisiae*, AP2 has been shown to facilitate the formation of the major class of endocytic vesicles (Myers and Payne, 2013; Lu et al., 2016). Moreover, we found that two genes were deleted in the transformant Cm699 (Figure 5A), which was probably a result of T-DNA integration employing double-strand break repair (Kleinboelting et al., 2015). These two genes may also be potential pathogenic genes that are not similar to known genes in fungi, and may be novel fungal pathogenic factors. T-DNA insertion into the promoter region of the potential pathogenicity gene was probably the main element responsible for the observed pathogenic defect in the transformant (Lee et al., 1990). In the transformant Cm854, the observed pathogenicity defect was presumably the reason for the disruption of the promoter region of a gene encoding Myosin I, which shares sequence homology with *MYO5* of *Pyricularia oryzae* (78%) by T-DNA integration. In *S. cerevisiae*, although the phenotype of *MYO5* deletion mutants did not change, growth and actin cytoskeletal organization were affected (Goodson et al., 1996). The observed pathogenicity defect in the transformant Cm1078 was possibly a result of a T-DNA insertion into the region of between two adjacent genes, which were simultaneously disrupted. One gene encoded a WD-repeat protein RBBP4, which also known as chromatin-remodeling factor RBAP48, shares sequence homology with MSl1 of *S. cerevisiae* (Ruggieri et al., 1989; Qian et al., 1993; Miao et al., 2020). Furthermore, the *MSl1* gene could suppress the defects in *S. cerevisiae* sporulation (Ruggieri et al., 1989). The other gene encoded a protein, which exhibited homology with the *XRN1* of *S. cerevisiae*, was not only involved in the termination of DNA transcription, but also in RNA degradation (Hsu and Stevens, 1993; Kim et al., 2004; Sharma et al., 2022).

In summary, the modified ATMT protocol is suitable for identifying novel genes required for the pathogenicity of the watermelon pathogen *C. magnum*. Six potential virulence genes were identified in *C. magnum*. These findings provide foundation for the pathogenic mechanism of *C. magnum.*

## Data availability statement

The datasets presented in this study can be found in online repositories. The names of the repository/repositories and accession number(s) can be found in the article/Supplementary material.

## Author contributions

QG and ZG conceptualized the study and designed the experiments. ZG performed the experiments, acquired and analyzed the data, and drafted the original manuscript. HW, CL, and QG reviewed and edited the manuscript. BP, BK, LL, HW, and QG supervised the work. All authors contributed to the article and approved the submitted version.

## Funding

This work was financially supported by Agricultural Science and Technology Innovation Program (CAAS-ASTIP-2022-ZFRI-09).

## Conflict of interest

The authors declare that the research was conducted in the absence of any commercial or financial relationships that could be construed as a potential conflict of interest.

## Publisher’s note

All claims expressed in this article are solely those of the authors and do not necessarily represent those of their affiliated organizations, or those of the publisher, the editors and the reviewers. Any product that may be evaluated in this article, or claim that may be made by its manufacturer, is not guaranteed or endorsed by the publisher.

## Footnotes

^1^ https://blast.ncbi.nlm.nih.gov/Blast.cgi

^2^ http://linux1.softberry.com/berry.phtml

^3^ https://github.com/BCH-RC/TDNAscan

^4^ http://www.ncbi.nlm.nih.gov
