# Supplementary material for: Identifying pathogenicity-related genes in the pathogen Colletotrichum magnum causing watermelon anthracnose disease via T-DNA insertion mutagenesis
Source: Front Microbiol. 2023 Jul 20;14:1220116. doi: 10.3389/fmicb.2023.1220116 (PMC10399754; doi:10.3389/fmicb.2023.1220116)
Supplement: Supplementary file 1 [file Table_1.docx]

**Table S1** Conditions used for thermal asymmetric interlaced polymerase chain reaction (TAIL-PCR).

| Primary PCR reaction | Secondary PCR reaction | Tertiary PCR reaction |
| --- | --- | --- |
| 92°C: 2 min | 94°C: 1 min | 94°C: 1 min |
| 95°C: 1 min | 94°C: 30 s** | 94°C: 30 s*** |
| 94°C: 30 s* | 62°C: 1 min** | 44°C: 1 min*** |
| 66°C: 1 min* | 72°C: 2 min** | 72°C: 2 min*** |
| 72°C: 2 min* | 94°C: 30 s** | 72°C: 8 min |
| 94°C: 30 s | 62°C: 1 min** |  |
| 30°C: 3 min | 72°C: 2 min** |  |
| Ramp to 72°C over 3 min | 94°C: 30 s** |  |
| 72°C: 2 min | 44°C: 1 min** |  |
| 94°C: 30 s** | 72°C: 2 min** |  |
| 65°C: 1 min** | 72°C: 8 min |  |
| 72°C: 2 min** |  |  |
| 94°C: 30 s** |  |  |
| 66°C: 1 min** |  |  |
| 72°C: 2 min** |  |  |
| 94°C: 30 s** |  |  |
| 44°C: 1 min** |  |  |
| 72°C: 2 min** |  |  |
| 72°C: 8 min |  |  |

a *, **, and*** represents ×5, and ×15, and ×30 cycles, respectively.

**Table S2.** Primer list used in validation.

| Name | Sequencea (5’ →3’) |
| --- | --- |
| 699L1F | AATGCTGGTTCGGAATGGC |
| 699r1R | TGCTTGCTCGTCTCCTCAA |
| 854r1F | TTGGTGCCTACAGCTTCGT |
| 854r2R | AACAGTGGTGCCTGACGAC |
| 1078r1F | GGTAGCGTGCCTTGTTGAC |
| 1078r2R | ACTGTAGACGATGGTGTTAGCA |
| 699r3F | GATGTTGATTAGGCCTTCCTGA |
| 699r3R | GACGAAGTTGGACTGGTACTT |
| 854r3F | CGTACTTGAGACCAAAGGGAAA |
| 854r3R | CTGCACAGCCTCAAGTCAT |
| 1078r3F | GACGATCTTGAACTCGACGGCA |
| 1078r3R | ATGTCGCCTGTGGGTAGT |
| R1 | GGCACTGGCCGTCGTTTTACAAC |
| L1 | GGGTTCCTATAGGGTTTCGCTCATG |
| AP1-F | GAGCGTGACGATGAAGAAGAC |
| AP1-R | CCTCCTGTTCCTTGACCTTGA |
| AP2-F | TGTCTGACGCTACCGCTAC |
| AP2-R | TGACTTCGCCACCATCCTC |
| AP3-F | GGTCCGCCAAGATGAAGATG |
| AP3-R | GCTCTGGAGATGACAACAAGG |
