# Supplementary material for: Identifying pathogenicity-related genes in the pathogen Colletotrichum magnum causing watermelon anthracnose disease via T-DNA insertion mutagenesis
Source: Front Microbiol. 2023 Jul 20;14:1220116. doi: 10.3389/fmicb.2023.1220116 (PMC10399754; doi:10.3389/fmicb.2023.1220116)
Supplement: Supplementary file 2 [file Data_Sheet_1.pdf]

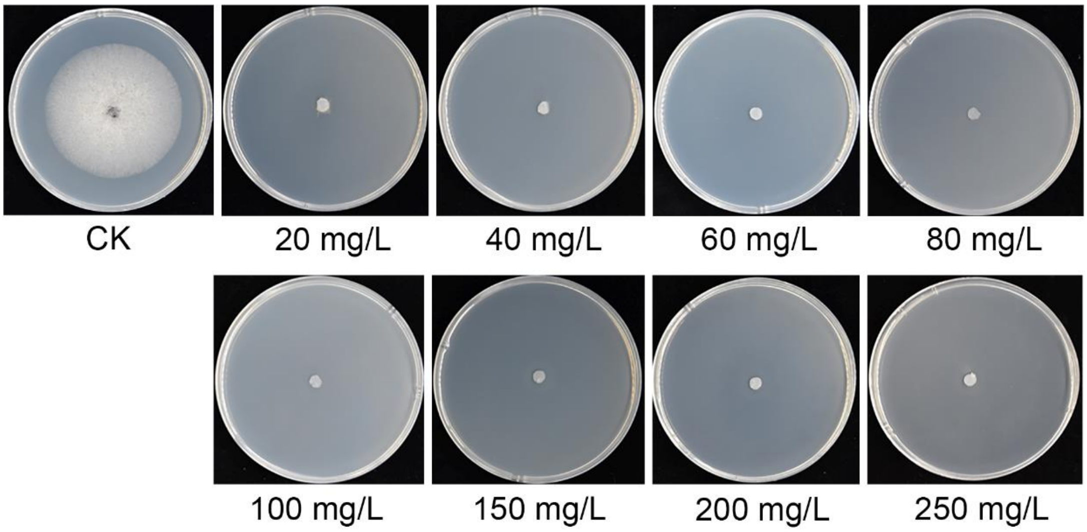

FIGURE S1. Effects of different concentrations of hygromycin B on the mycelial growth of *Colletotrichum magnum*. Mycelial plugs (0.5 cm in diameter) from the edge of freshly growing cultures of the wild type strain CAASZK4 were placed on PDA containing different concentrations of hygromycin B. The pictures were taken at 5 dpi.

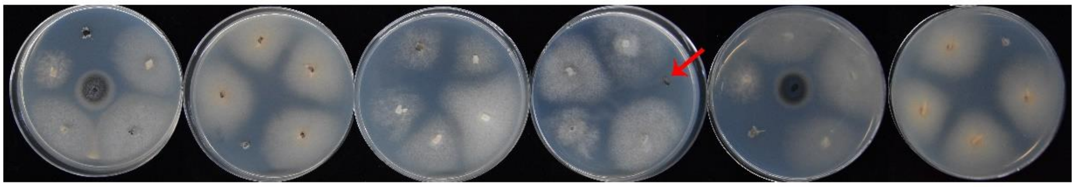

FIGURE S2. Mitotic stability of *Colletotrichum magnum* transformants. After subcultring 5 times on PDA in the absence of antibiotic, the randomly selected transformant was transferred to PDA containing 70 mg/L hygromycin B. All the randomly selected transformants grew normally whereas the CAASZK4 strain (arrow) did not. The pictures were taken at 3 dpi.

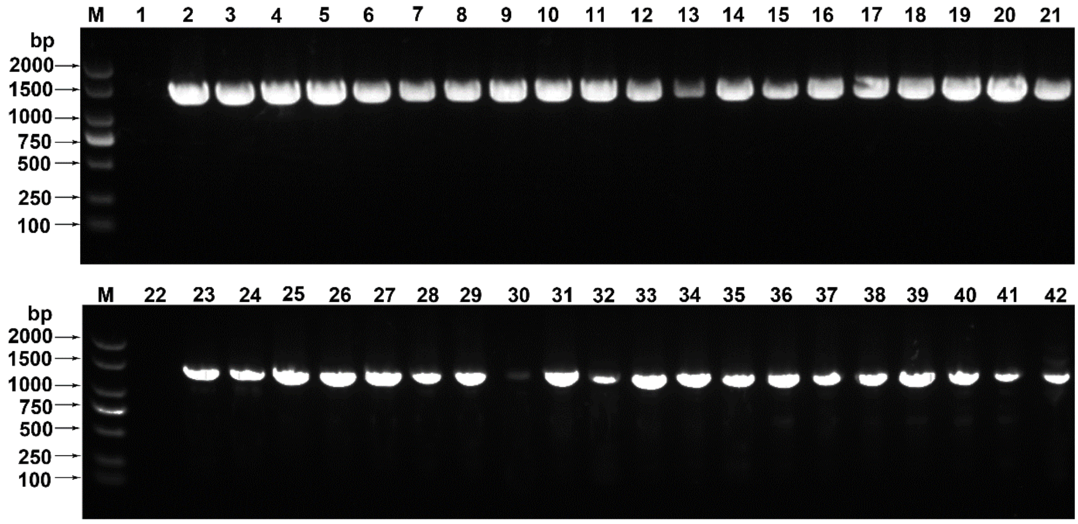

FIGURE S3. Confirmation of *hph* gene integration in *Colletotrichum magnum* transformants. M: 2 kb DNA marker. 1,22: wild type untransformed control. 2─22, 23─42: random transformants.

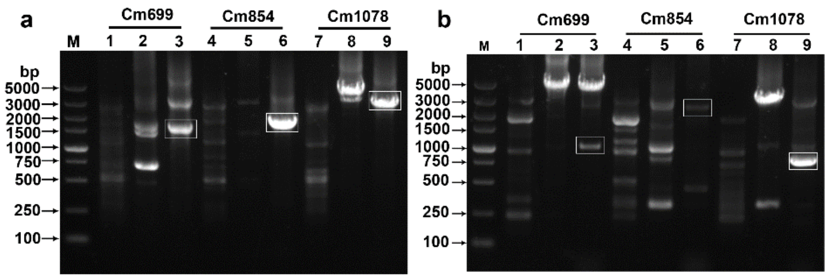

FIGURE S4. TAIL-PCR amplification of T-DNA flanking sequences of three transformants (Cm699, Cm854 and Cm1078). Sequences flanking the left border of the T-DNA (a). Sequences flanking the right border of the T-DNA (b).1, 4, 7: PCR products from the first rounds of TAIL-PCR amplification. 2, 5, 8: PCR products from the second rounds of TAIL-PCR amplification. 3, 6, 9: PCR products from the third rounds of TAIL-PCR amplification. M: 5 kb DNA marker. Rectangle indicate successful PCR amplification of a flanking region of T-DNA in the three transformants.

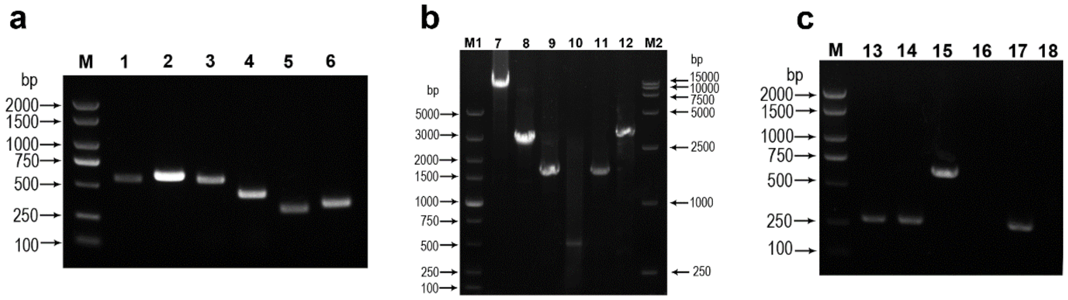

FIGURE S5. The validation of the TISs, the patterns of T-DNA integration, and deleted gene. (a) The validation of six TISs; (b) Amplification sequences flanking the left and right border of the T-DNA of transformants by nested PCR; (c) The validation of deleted gene. 1, 2: left and right TISs of Cm699, 1: using the primer pairs 699L1F + L1, 2: using the primer pairs 699r1R + R1; 3, 4: left and right TISs of Cm854, 3: using the primer pairs 854r1F + R1, 4: using the primer pairs 854r2R + R1; 5, 6: left and right TISs of Cm1078, 5: using the primer of pairs 1078r1F + R1, 6: 1078r2R + R1. 7, 8: Amplification sequences flanking the left and right border of the T-DNA of Cm699 using two pairs of primers 699L1F + 699r1R and 699r3F + 699r3R, 7: WT strains, 8: Cm699; 9, 10: Amplification sequences flanking the left and right border of the T-DNA of Cm854 using two pairs of primers 854r1F + 854r2R and 854r3F + 854r3R, 9: Cm854, 10: WT strains; 11, 12: Amplification sequences flanking the left and right border of the T-DNA of Cm1078 using two pairs of primers 1078r1F + 1078r2R and 1078r3F + 1078r3R, 11: WT strains, 12: Cm1078. 13, 14: Amplification of *EVM0012133* using the primer pairs AP3-F + AP3-R, 15, 16: Amplification of *EVM0008660* using the primer pairs AP2-F + AP2-R, 17, 18: Amplification of *EVM0004795* using the primer pairs AP1-F + AP1-R, 13, 15, 17: WT strains, 14, 16, 18: Cm699. M: 2 kb DNA marker; M1: 5 kb DNA marker; M2: 15 kb DNA marker.

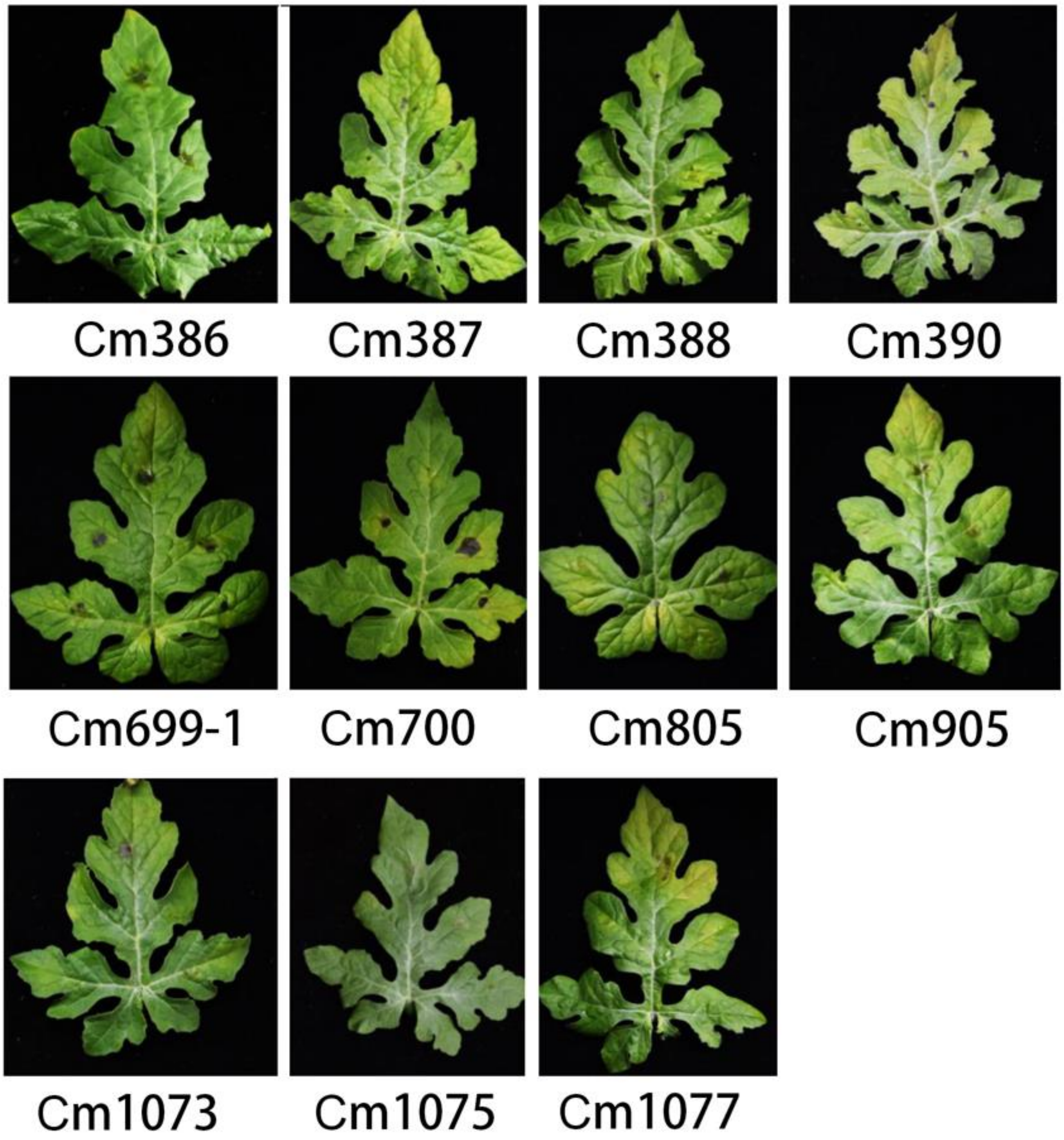

FIGURE S6 Symptoms of watermelon leaves (*Citrullus. lanatus* cv. Hongheping) induced by 11 transformants of C. magnum
